# Cervical injection as an alternative to the utero-ovarian ligament for mapping pelvic sentinel lymph node in early-stage ovarian cancer

**DOI:** 10.1007/s00404-025-07984-x

**Published:** 2025-04-05

**Authors:** Víctor Lago, Iria Rey, Marta Arnáez, Pablo Padilla-Iserte, Luis Matute, Marta Gurrea, Sara Moner, Pilar Bello, Santiago Domingo

**Affiliations:** 1 Gynecologic Oncology Unit, University Hospital La Fe, Avinguda de Fernando Abril Martorell, 106, Tower F, 3rd Floor, 46026 Valencia, Spain; 2 Instituto de Investigación Sanitaria La Fe (IISLAFE), Valencia, Spain; 3 Department of Medicine, CEU Cardenal Herrera University, Comunitat Valenciana, Valencia, Spain; 4 Department of Obstetrics, Gynecology and Pediatrics, University of Valencia, Valencia, Spain; 5 Department of Obstetrics and Gynecology, General University Hospital of Valencia, Valencia, Spain; 6 Department of Nuclear Medicine, University Hospital La Fe, Valencia, Spain

**Keywords:** Ovarian cancer, Early-stage, Sentinel lymph node, Injection site, Indocyanine green, Technetium-99 m

## Abstract

**Purpose:**

In early-stage ovarian cancer, sentinel lymph node (SLN) mapping using double injection into the utero-ovarian and infundibulo-pelvic ligaments has been postulated. Cervical injection, commonly used in other gynaecologic tumors, may provide a simpler alternative to utero-ovarian injection for pelvic-SLN detection. This study aims to demonstrate whether cervical and utero-ovarian injections drain to the same pelvic SLN using different tracers for each injection site: technetium-99m (^99m^Tc) at cervix and indocyanine green into the utero-ovarian ligament.

**Methods:**

This prospective trial enrolled endometrial cancer patients scheduled for SLN biopsy from July 2023 to May 2024. Each hemipelvis was considered a case. ^99m^Tc was injected at the cervix preoperatively. If ^99m^Tc migration occurred, indocyanine green was injected into the utero-ovarian ligament intraoperatively. Concordance of migration was determined in those hemipelvis with both ^99m^Tc-cervical and indocyanine green utero-ovarian migration.

**Results:**

Seventeen patients (34 hemipelvis) were included. Migration from both injection sites occurred in 17 hemipelvis, identifying the same pelvic-SLN in all cases, being the concordance rate of 100%. Migration of ^99m^Tc or indocyanine green from cervical injection was detected in 91.2% (95% CI 81.6–100%), whereas migration of indocyanine green injection from the utero-ovarian ligament was detected in 73.9% (95% CI 56–91.9%); these detection rates were not significantly different (*p* = 0.077).

**Conclusions:**

Lymphatic migration from the cervix to the pelvis seems to be comparable to the migration from the utero-ovarian ligament to the pelvis, with both pathways converging at the same SLN.

## What does this study add to the clinical work

**Table:** 

Cervical injection is the standard technique used for pelvic-SLN detection in early-stage cervical and endometrial cancer. However, in early-stage ovarian cancer, sentinel lymph node (SLN) mapping using double injection into the utero-ovarian and infundibulo-pelvic ligaments has been postulated, but the technique is still under investigation partly due to the complexity of utero-ovarian and infundibulo-pelvic ligaments or stumps injections. Cervical injection should be considered as an easier and more reproducible technique for pelvic-SLN detection compared with current practices, since both cervical and utero-ovarian injections sites appear to be equivalent as the same pelvic SLN was identified in all the cases (100% concordance rate).	

## Introduction

Sentinel lymph node (SLN) mapping is a well-established technique for nodal assessment in most gynaecologic malignancies, including vulvar, cervical and endometrial cancers [1–4]. Nevertheless, its application in epithelial ovarian cancer remains under investigation due to the complexity of ovarian lymphatic drainage and the variety of methodological approaches that are available.

Ovarian lymphatic drainage occurs bidirectionally: through the utero-ovarian ligament to the pelvis field and through the infundibulo-pelvic ligament to the para-aortic field [5–7]. This drainage becomes unidirectional after adnexectomy, necessitating a double injection into utero-ovarian and infundibulo-pelvic ligaments or stumps for effective SLN detection, as reported in numerous trials published to date, showing encouraging results [8–16]. However, technical complexity hinders the reproducibility of this technique, especially regarding the utero-ovarian ligament injection site, for which an overall pelvic-SLN-detection rate of 59.5% (95% CI 50.2–68.1%) has been reported [17].

The theoretical pelvic lymphatic pathway primarily drains the pelvic organs through a lateral route towards the obturator nodal group to the middle and lateral chains of the external iliac nodes, and through a medial or hypogastric route along the hypogastric vessels, with both routes converging at the junctional nodes and ascending to the common iliac and para-aortic nodes. Tumours of the endometrium, cervix and ovary can spread along these pathways [5–7]. A recent study suggests that tracers injected into the utero-ovarian ligament and the cervix could migrate through equivalent lymphatic drainage routes [18]. Given widespread cervical injection for pelvic SLN detection in other gynaecologic tumours, it could be a more simple and reproducible method compared with utero-ovarian ligament injections for SLN technique in early-stage ovarian cancer patients [1–4] representing a real strategy to improve the low detection rate of pelvic-SLN in ovarian cancer [17]. On the other hand, the lymphatic spread of ovarian cancer can also skip the pelvic nodes and follow the ovarian vessels along the infundibulo-pelvic ligament to reach the para-aortic or para-caval lymph nodes [5–7]. Therefore, an accompanying tracer injection into the infundibulo-pelvic ligament is mandatory to detect para-aortic metastases.

This prospective study aimed to determine and confirm whether both cervical and utero-ovarian ligament tracer injections drained to the same sentinel pelvic lymph node. To answer this question, two different tracers were injected into two different locations to determine whether both tracers migrated to the same pelvic SLN: technetium-99 m (^99m^Tc) was injected into the cervix and indocyanine green (ICG) was injected into the utero-ovarian ligament.

## Materials and methods

We performed a prospective observational single-centre trial from July 2023 to May 2024, including patients who had been diagnosed with endometrial tumours for which SLN biopsy had been scheduled. Patients with a history of previous vascular pelvic surgery or lymphadenectomy, lymphoma or radiotherapy (pelvic or para-aortic fields) and/or a previous allergic reaction to colloids or indocyanine green were not considered for inclusion. This study obtained ethics committee approval (Local ID cod: 2022-790-1) and all patients were prospectively enrolled after giving informed consent. In accordance with the journal’s guidelines, we will provide our data for independent analysis by a selected team by the Editorial Team for the purposes of additional data analysis or for the reproducibility of this study in other centres if such is requested.

**Fig. 1:**
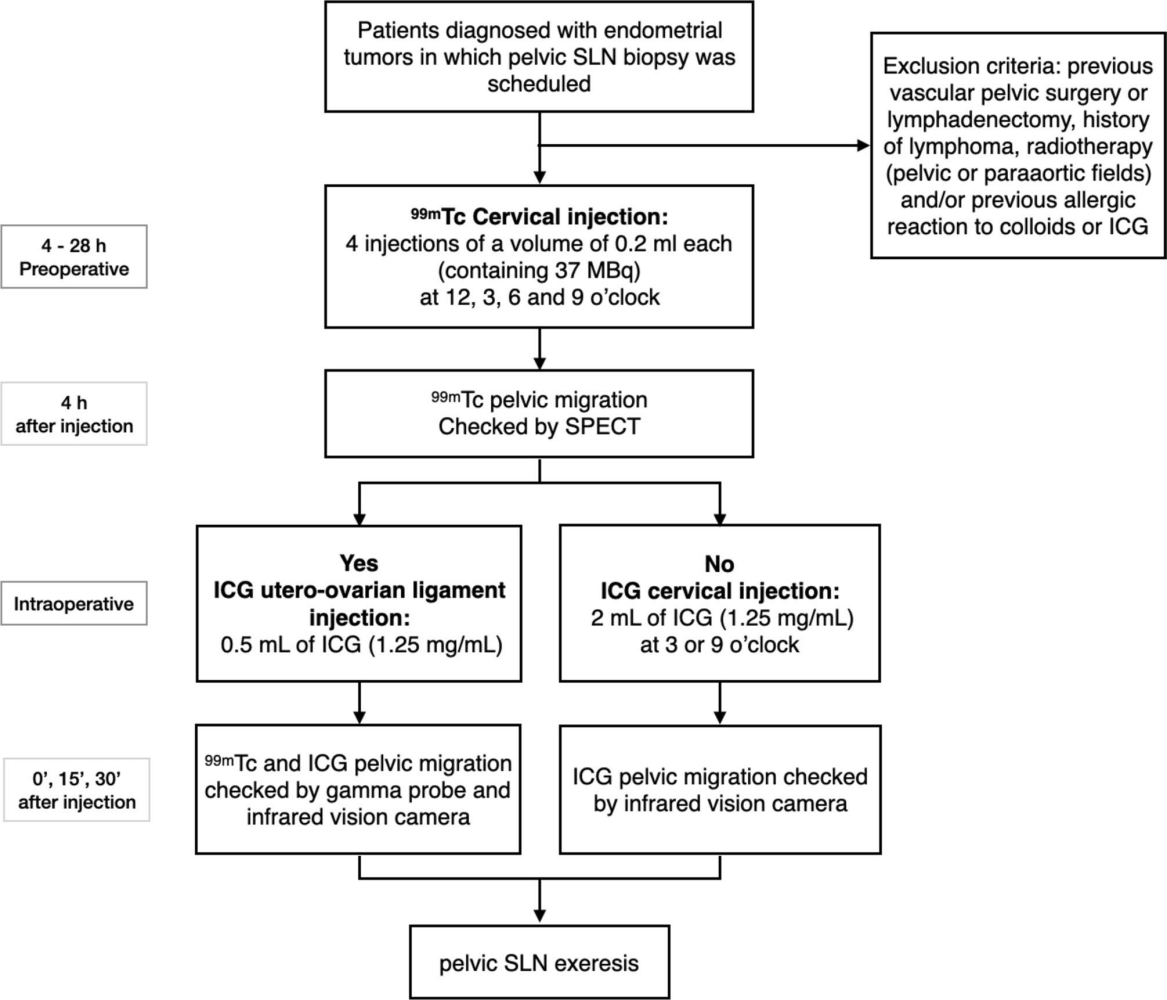
Sentinel lymph node technique scheme for each hemipelvis. *SLN* sentinel lymph node, *ICG* indocyanine green, ^*99m*^*Tc* technetium-99 m, *SPECT* single-photon emission computed tomography

Each hemipelvis from a patient was considered a distinct case for the purpose of this study such that each patient provided up to two cases. The sequence of injection is described in the flow chart in Fig. 1.

**Fig. 2:**
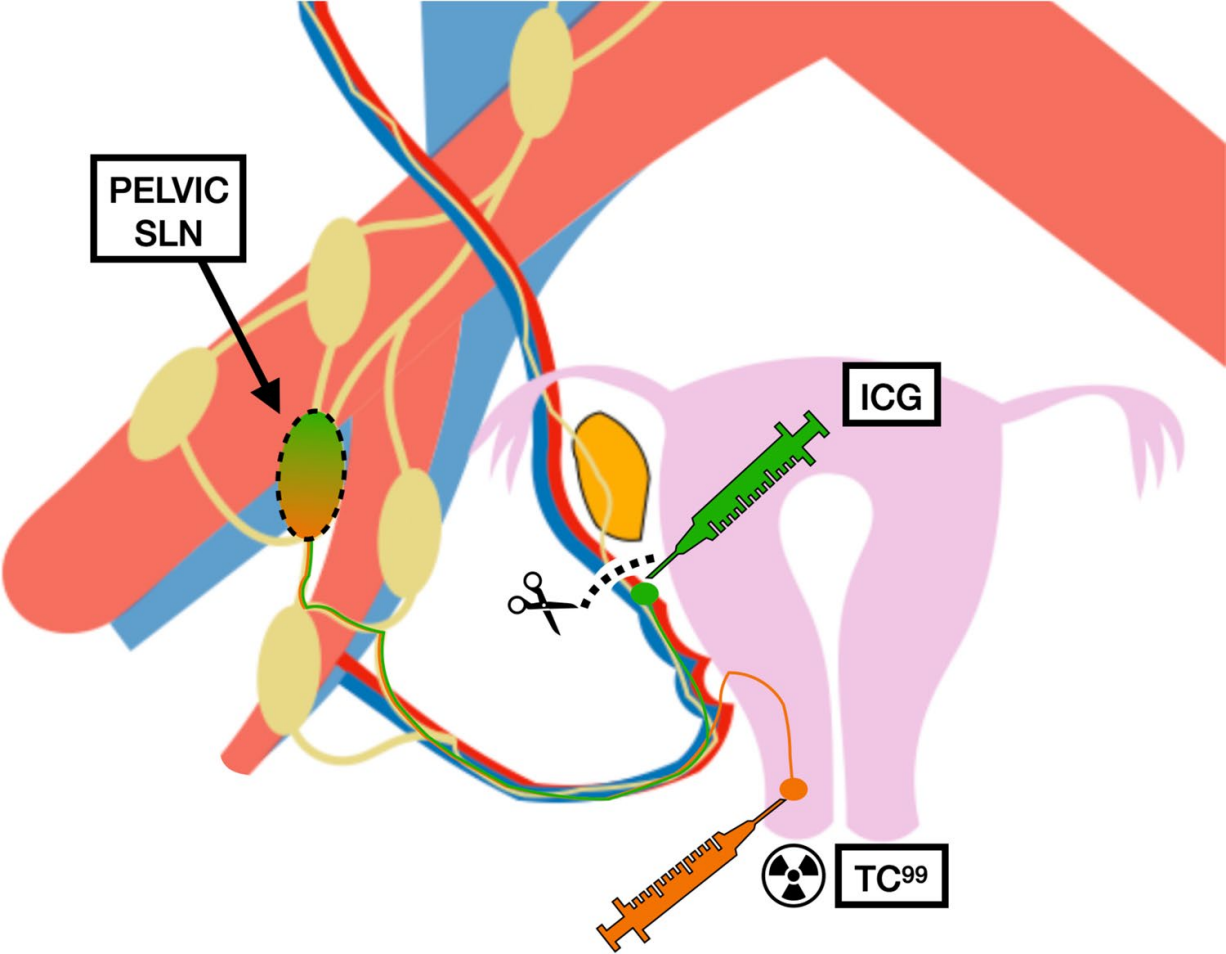
Scheme of tracer injection into the cervix and utero-ovarian ligament. *SLN* sentinel lymph node, *ICG* indocyanine green, ^*99m*^*Tc* technetium-99 m

^99m^Tc injection into the cervix was performed as follows: 4–28 h before the surgical procedure as an outpatient, four 0.2 ml injections, each containing 37 MBq of ^99m^Tc, were administered at 12, 3, 6 and 9 o’clock at 1 cm depth. At 4 h after the injection, a single-photon emission computed tomography scan (SPECT) was performed before surgery to identify the exact location of the SLN to which the tracer migrated to guide its detection during surgery. In those cases in which the migration of ^99m^Tc was observed in the SPECT or intraoperatively guided by the gammaprobe, an indocyanine green injection at the utero-ovarian ligament was performed as follows: first, the utero-ovarian ligament was sealed and sectioned to convert the migration of the ovary into a unilateral flow leading to the pelvic field and then a 0.5 mL injection of indocyanine green (1.25 mg/mL) was performed under direct vision. The concordance of migration could only be determined in hemipelvis with both ^99m^Tc migration from the cervix and indocyanine green migration from the utero-ovarian ligament (Fig. 2).

In those hemipelvis in which there was no migration of ^99m^Tc from the cervix, the standard SLN technique for endometrial cancer patients as per protocol in our centre: during surgery, 2 mL of indocyanine green (1.25 mg/mL) was injected into the cervix. Those hemipelvis with no migration of ^99m^Tc from the cervix or no migration of indocyanine green from the utero-ovarian ligament was excluded from the concordance analysis.

In those hemipelvis with ^99m^Tc migration, the cervix and the pelvic field were checked at 0, 15 and 30 min after indocyanine green injection with the gammaprobe and the infrared vision camera. Detection was performed using gentle and minimal dissection, guided by the acoustic signal of the gamma probe and following the lymphatic vessels dyed with indocyanine green arising from the injection point at the utero-ovarian ligament by direct vision. Regarding the use of ^99m^Tc as a tracer, any lymph node with a count markedly greater than ten times the background level was considered a SLN. The first indocyanine green-stained node following the path of the lymphatic vessels from the injection site was considered a SLN. All SLNs that were retrieved were classified according to the anatomical region in which they were located, considering the concordance (if present) between both markers.

After SLN detection, each patient underwent the standard surgical procedure that had been preoperatively determined according to their tumour type and stage by the hospital’s tumour board; this study did not deviate for any case from the standard surgery that had been determined in this way and proposed to the patient.

The primary outcome of this study was to determine the percentage of concordance of two different injection sites for pelvic-SLN detection using a different tracer at each injection site (^99m^Tc injection in the cervical versus indocyanine green injection in the utero-ovarian ligament). The secondary outcomes were to determine the global detection rate and the detection rate for each injection site and tracer.

### Statistical analysis

Descriptive statistics are reported as absolute frequency (percentage) for categorical variables and as mean, standard deviation (SD) and range for continuous variables. The probability of detecting the same pelvic SLN (concordance rate) and the probability of detecting a different or additional SLN (discordance rate) in each hemipelvis were compared using McNemar’s exact test. Statistical significance was defined as *p* < 0.05 (two-tailed). Based on the McNemar’s exact test statistics, we needed to detect the pelvic SLN with both techniques in 15 hemipelvis to have a power of 95% to reject the null hypothesis that the probability of detecting only the same SLN (proportion of hemipelvis with the same detected SLN) is equal to the probability of detecting only different pelvic SLNs (proportion of hemipelvis with two different detected SLNs). The sample size was estimated by assuming that 90% of the hemipelvis would show the detection of the same SLN, whereas 10% of the hemipelvis would show the detection of different SLNs. We planned to enrol patients until a total of 16 hemipelvis in which pelvic SLNs were detected using both techniques.

## Results

A total of 17 consecutive patients (34 hemipelvis) who were diagnosed with endometrial cancer between July 2023 and May 2024 met the inclusion criteria and were enrolled. The clinical and surgical characteristics are summarised in Table 1. The mean age was 61.7 ± 13.3 (range: 33–84) years, and the average body mass index (BMI) was 28.3 ± 6.5 (range 18.3–41.7) kg/m^2^.

**Table 1:** Clinical and surgical characteristics

Age [mean ± SD (range)]; year	61.7 ± 13.3 (33–84)
BMI [mean ± SD (range)]; kg/m^2^	28.3 ± 6.5 (18.3–41.7)
Approach; *n* (%)	
Laparoscopy	1 (5.9)
Robotic	16 (94.1)
Estimated blood loss [mean ± SD]; ml	84.4 ± 43.7 (50–200)
Surgical time [mean ± SD (range)]; min	193.4 ± 47.7 (128–310)
Intraoperative complications; n (%)	0 (0)
Hospitalisation time [mean ± SD (range)]; day	1.5 ± 0.8 (1–4)
Complications (Clavien–Dindo); *n* (%)	
No	16 (94.1)
Grade I	1 (5.9)
Histotype, *n* (%)	
Endometrioid	12 (70.6)
Serous	3 (17.6)
EIN	2 (11.8)
FIGO grade, *n* (%)	
G1	10 (58.8)
G2	1 (5.9)
G3	4 (23.5)
NA	2 (11.8)
Molecular classification, *n* (%)	
POLEmut	1 (5.9)
MMRD/MSI	5 (29.4)
NSMP	5 (29.4)
p53abn	4 (23.5)
NA	2 (11.8)
LVI, *n* (%)	
No	15 (88.2)
Yes	2 (11.8)
FIGO final stage, *n* (%)	
IA	9 (52.9)
IB	3 (17.6)
IIIC	3 (17.6)
NA	2 (11.8)

*SD* standard deviation, *BMI* body mass index, *EIN* endometrial intraepithelial neoplasia, *POLEmut* POLE mutation, *MMRD/MSI* mismatch repair deficient/microsatellite instability, *NSMP* no specific molecular profile, *p53abn* p53 abnormal, *LVI* lymphovascular invasion, *FIGO* International Federation of Gynecology and Obstetrics

The surgical approach was minimally invasive in all cases: robotic surgery was performed in 94.1% of the cases and laparoscopy was performed in 5.9% of the cases. The mean operating time was 193.4 ± 47.7 min. No intra-operative complications were reported, and the mean duration of hospitalisation was 1.5 ± 0.8 days (range 1–4).

Final histology was endometrioid endometrial cancer in 70.6% of the cases, serous endometrial cancer in 17.6% of the cases and endometrial intraepithelial neoplasia (EIN) in 11.8% of the cases. Excluding EIN patients, the tumour grade was G1 in 58.8%, G2 in 5.9% and G3 in 23.5% of the patients. Regarding the molecular classification, 5.9% of the cases had a POLE mutation (POLEmut), 29.4% of the cases had mismatch repair deficient/microsatellite instability (MMRD/MSI), 23.5% of the cases had a p53 abnormal variant (p53abn) and 29.4% of the cases showed no specific molecular profile (NSMP). The final International Federation of Gynecology and Obstetrics (FIGO) stage was IA in 52.9%, IB in 17.6% and IIIC in 17.6% of the cases.

^99m^Tc was injected bilaterally into the cervix before surgery in all 17 patients, and its migration to a pelvic SLN was detected in 23/34 hemipelvis by SPECT, giving a cervical ^99m^Tc detection rate of 67.7% (95% CI 51.9–83.4%). Bilateral migration of ^99m^Tc occurred in 8/17 patients (47.1%; 95% CI 23.3–70.8%).

In all the 23 hemipelvis in which pelvic SLN was detected by ^99m^Tc, indocyanine green was injected into the utero-ovarian stump; ICG migration was detected in 17/23 hemipelvis, which gave a utero-ovarian indocyanine green detection rate of 73.9% (95% CI 56–91.9%); bilateral migration occurred in 5/8 patients (62.5%; 95% CI 29–96%).

In the 11 hemipelvis in which SLN was not detected by ^99m^Tc, indocyanine green was injected into the cervix ipsilaterally, leading to the detection of an SLN in 8/11 hemipelvis (72.7%; 95% CI 46.4–99%). Considering the use either tracer (^99m^Tc or indocyanine green), the rate of SLN detection via the cervical injection technique was 91.2% (31/34 hemipelvis, 95% CI 81.6–100%), and this occurred bilaterally in 14/17 patients (82.4%; 95% CI 64–100%). There were no significant differences between the cervical-detection rate (91.2%; 95% CI 81.6–100%) and the utero-ovarian-detection rate (73.9%; 95% CI 56–91.9%; *p* = 0.077).

Among the 17 hemipelvis in which ^99m^Tc from cervical injection and indocyanine green from utero-ovarian-stump injection successfully migrated, both techniques identified the same pelvic SLN in all cases, with a concordance rate of 100% (Table 2).

**Table 2:** Sentinel lymph node procedure

Pelvic SLN removed ([mean ± SD (range)]; nodes	1 ± 0.5 (0–2)
SLN detection rate; *n* (%)	
Cervical (^99m^Tc) detection rate (n34 hemipelvis)	23 (67.7)
Cervical (^99m^Tc) bilateral detection rate (n17 patients)	8 (47.1)
Utero-ovarian (ICG) detection rate (n23 hemipelvis)	17 (73.9)
Utero-ovarian (ICG) bilateral detection rate (n8 patients)	5 (62.5)
Global (cervical ^99m^Tc or ICG) detection rate (n34 hemipelvis)	31 (91.2)
Global (cervical ^99m^Tc or ICG) bilateral detection rate (n17 patients)	14 (82.4)
Concordance location between cervical (^99m^Tc) and utero-ovarian (ICG) SLN detection; *n* (%) (n17 hemipelvis)	17 (100)
SLN location; *n* (%) (n17 hemipelvis)	
Right common iliac vessel	0 (0)
Left common iliac vessel	1 (5.9)
Right bifurcation iliac vessel	1 (5.9)
Left bifurcation iliac vessel	0 (0)
Right external iliac vessel	3 (17.6)
Left external iliac vessel	3 (17.6)
Right obturator	5 (29.4)
Left obturator	2 (11.8)
Right internal iliac vessel	2 (11.8)
Left external iliac vessel	0 (0)
SLN metastases rate; *n* (%) (n17 patients)	3 (17.6)
SLN ultrastaging; *n* (%) (n3 patients)	3 (100)

*SD* standard deviation, ^*99m*^*Tc* technetium-99 m, *ICG* indocyanine green, *SLN* sentinel lymph node

There were three (9%) hemipelvis in which SLN detection failed: in two of these hemipelvis, a lymphadenectomy was performed, whereas in the other case, lymphadenectomy was not performed due to the nature of the patient’s disease (EIN). The mean number of harvested SLNs was 1 ± 0.5 (range: 0–2). In the three patients (3/17; 17.6%) with lymph node metastases, these were detected in a SLN by ultrastaging (100%); metastases were unilateral in one case and bilateral in two cases, and they included one macrometastasis, one micrometastasis and three ITCs (5 hemipelvis). All lymph node metastases detected were present in an SLN that was identified by both tracers (^99m^Tc from the cervix and ICG from the utero-ovarian ligament).

## Discussion

To date, the published studies investigating SLN in early-stage ovarian cancer have explored different injection sites such as the infundibulo-pelvic and utero-ovarian ligaments [8–15], the hilum of the ovary [16] and the ovarian cortex [8]. Based on the bidirectional drainage of the ovary towards the pelvic and para-aortic areas [5–7] which is interrupted when an adnexectomy is performed, injection into the utero-ovarian and infundibulo-pelvic ligaments or stumps to evaluate the pelvic and para-aortic fields, respectively, are the most widely explored injection sites, showing SLN-detection rates of up to 92.9% (95% CI 73.7–100%; *I*^2^ = 80.5%; *p* = 0.61) for the ligament injection when compared with the rates of other injection sites [19].

Nevertheless, if the SLN-detection rate is calculated separately for each field, the pelvic-SLN detection rate decreases to 59.5% (95% CI 50.2–68.1%) according to a recent meta-analysis [17]. Based on previous experience [9, 10], there are some concerns about utero-ovarian ligament injection. Sub-peritoneal injection in the same surgical field can lead to spillage of the tracer, thus hindering the localisation of the pelvic lymph node and accounting for its lower detection rate. As cervical injections for pelvic-SLN detection are widely used in gynaecological oncology, their use would facilitate the implementation of the technique in early-ovarian stage SLN mapping. According to our results, cervical and utero-ovarian tracer injection sites appear to be equivalent techniques for pelvic-SLN detection since both sites identified the same pelvic SLN in each case (100% concordance rate), corroborating the theory proposed by Uccella et al. [18].

The overall SLN detection rate for the cervical injection technique (using ^99m^Tc or indocyanine green) was high, with migration observed in 31/34 hemipelvis (91.2%, 95% CI 81.6–100%). On the other hand, the pelvic-SLN detection rate for the utero-ovarian injection technique was lower than cervix, being 73.9% (95% CI 56–91.9%; *p* = 0.077). Despite not reaching statistical significance, this difference is in line with what was reported by Uccella et al. [18] who obtained a higher SLN-detection rate of 86.1% for cervical injection compared with a 52.8% SLN-detection rate for utero-ovarian injection (*p* = 0.0004). These SLN-detection rates from the cervix are similar to those obtained in endometrial cancer studies [4]. The observed global cervical-detection rate (using ^99m^Tc or indocyanine green) highlights the fact that combined usage of both tracers could improve the pelvic detection rate in cervical injections and should, therefore, be considered in future investigations.

Anatomic studies have shown a low migration rate (50%) from the ovary to the pelvis through the utero-ovarian ligament [20]. Considering utero-ovarian injection limitations, performing cervical injection for pelvic mapping is a simplification of the technique that would improve pelvic-SLN-detection rates, as gynaecological oncologists are familiar with this injection technique after years of implementing it in endometrial and cervical cancer.

However, although we observed in this study a concordance rate of migration of 100% between cervix and utero-ovarian ligament, a reasonable question is whether the true SLN was identified. Our results clarify this question as the lymph node metastases that were found were only present in the SLN where there was a concordance of the two tracers that migrated from the cervix and the utero-ovarian ligament.

### Strengths and weakness

Our study was strengthened by its prospective design. Nevertheless, some limitations must be considered. This study was carried out in patients with endometrial cancer, with the aim of conducting an anatomic study of the lymphatic drainage of the uterus and the adnexa that could be applied to ovarian cancer. Although the type of cancer is unlikely to change drainage pathways, these results should be interpreted with caution. In addition, the use of two different tracers for each injection point limits the comparison of detection rates between the injection sites. Since concordance of migration could only be determined in hemipelvis with both ^99m^Tc migration from the cervix and indocyanine green migration from the utero-ovarian ligament, the intra-operative indocyanine green was only performed in those cases in which the migration of ^99m^Tc was observed, which makes the sample size of both groups differ, also limiting the comparability of the sample. Small sample size may also represent a limitation for the present exploratory study, and its findings must be confirmed with subsequent clinical trials.

## Conclusions

Lymphatic migration from the cervix to the pelvis seems to be equivalent to utero-ovarian ligament migration to the pelvis, converging to the same SLN. Performing cervical injection for pelvic mapping, which is a validated and less challenging technique that is widely used in gynaecological cancers, could improve pelvic-SLN-detection rates in early-stage ovarian cancer, allowing progress in future research towards the implementation of the SLN technique.

## Data Availability

Data is provided within the manuscript file, and we will provide additional data for independent analysis by a selected team by the Editorial Team if requested.

## References

[1] Levenback CF, Ali S, Coleman RL et al (2012) Lymphatic mapping and sentinel lymph node biopsy in women with squamous cell carcinoma of the vulva: a gynecologic oncology group study. J Clin Oncol 30(31):3786–3791 22753905 10.1200/JCO.2011.41.2528 PMC3478573

[2] Lécuru F, Mathevet P, Querleu D et al (2011) Bilateral negative sentinel nodes accurately predict absence of lymph node metastasis in early cervical cancer: results of the SENTICOL study. J Clin Oncol 29(13):1686–1691 21444878 10.1200/JCO.2010.32.0432

[3] Daraï E, Dubernard G, Bats AS et al (2015) Sentinel node biopsy for the management of early stage endometrial cancer: long-term results of the SENTI-ENDO study. Gynecol Oncol 136(1):54–59 25450151 10.1016/j.ygyno.2014.09.011

[4] Holloway RW, Abu-Rustum NR, Backes FJ et al (2017) Sentinel lymph node mapping and staging in endometrial cancer: a Society of Gynecologic Oncology literature review with consensus recommendations. Gynecol Oncol 146(2):405–415 28566221 10.1016/j.ygyno.2017.05.027 PMC6075736

[5] Paño B, Sebastià C, Ripoll E et al (2015) Pathways of lymphatic spread in gynecologic malignancies. Radiographics 35(3):916–945 25969940 10.1148/rg.2015140086

[6] Park JM, Charnsangavej C, Yoshimitsu K et al (1994) Pathways of nodal metastasis from pelvic tumors: CT demonstration. Radiographics 14(6):1309–1321 7855343 10.1148/radiographics.14.6.7855343

[7] Kleppe M, Kraima AC, Kruitwagen RFPM et al (2015) Understanding lymphatic drainage pathways of the ovaries to predict sites for sentinel nodes in ovarian cancer. Int J Gynecol Cancer 25(8):1405–1414 26397066 10.1097/IGC.0000000000000514 PMC5106084

[8] Hassanzadeh M, Hosseini Farahabadi E, Yousefi Z et al (2016) Lymphatic mapping and sentinel node biopsy in ovarian tumors: a study using intra-operative Tc-99m-Phytate and lymphoscintigraphy imaging. J Ovarian Res 9(1):55 27604260 10.1186/s13048-016-0265-4 PMC5013627

[9] Lago V, Bello P, Montero B et al (2019) Clinical application of the sentinel lymph node technique in early ovarian cancer: a pilot study. Int J Gynecol Cancer 29(2):377–381 30718316 10.1136/ijgc-2018-000049

[10] Lago V, Bello P, Montero B et al (2020) Sentinel lymph node technique in early-stage ovarian cancer (SENTOV): a phase II clinical trial. Int J Gynecol Cancer 30(9):1390–1396 32448808 10.1136/ijgc-2020-001289 PMC7497563

[11] Agustí N, Vidal-Sicart S, Paredes P et al (2023) Mapping sentinel lymph nodes in early-stage ovarian cancer (MELISA) trial—a further step towards lymphadenectomy replacement. Gynecol Oncol 179:145–151 37980769 10.1016/j.ygyno.2023.11.007

[12] Nero C, Bizzarri N, Di Berardino S et al (2023) Sentinel-node biopsy in apparent early stage ovarian cancer: final results of a prospective multicentre study (SELLY). Eur J Cancer 196:113435 38006759 10.1016/j.ejca.2023.113435

[13] Laven P, Kruitwagen R, Zusterzeel P et al (2021) Sentinel lymph node identification in early stage ovarian cancer: is it still possible after prior tumor resection? J Ovarian Res 14:1–6 34645514 10.1186/s13048-021-00887-w PMC8513191

[14] Buda A, Passoni P, Corrado G et al (2017) Near-infrared fluorescence-guided sentinel node mapping of the ovary with Indocyanine green in a minimally invasive setting: a feasible study. J Minim Invasive Gynecol 24:165–170 27670732 10.1016/j.jmig.2016.09.006

[15] Kleppe M, Brans B, Van Gorp T et al (2014) The detection of sentinel nodes in ovarian cancer: a feasibility study. J Nucl Med 55(11):1799–1804 25332439 10.2967/jnumed.114.144329

[16] Angelucci M, Corrado G, Mancini E et al (2016) Laparoscopic indocyanine green sentinel lymph node mapping in early ovarian cancer: a pilot study and review of the literature. Ital J Gynaecol Obstet 28:23–28

[17] Rey I, Lago V, Arnáez M et al (2024) Key issues in diagnostic accuracy of sentinel lymph node biopsy in early-stage ovarian cancer: systematic review and meta-analysis. Int J Gynecol Cancer 34(11):1787–1794 39414311 10.1136/ijgc-2024-005970

[18] Uccella S, Garzon S, Bosco M et al (2022) Cervical versus utero-ovarian ligament injection of the tracer for the pelvic sentinel lymph node mapping in gynecologic oncology: a prospective observational study. Gynecol Obstet Invest 87(3–4):242–247 35584619 10.1159/000525126

[19] Agusti N, Viveros-Carreño D, Grillo-Ardila C et al (2023) Sentinel lymph node detection in early-stage ovarian cancer: a systematic review and meta-analysis. Int J Gynecol Cancer 33(10):1493–1501 37487662 10.1136/ijgc-2023-004572

[20] Murris F, Weyl A, Ouldamer L et al (2024) Contribution of the cadaveric recirculation system in the anatomical study of lymphatic drainage of the ovary: applications in the management of ovarian cancer. Surg Radiol Anat 46(8):1155–1164 38900203 10.1007/s00276-024-03406-w

